# What Are We Actually Feeding ICU Patients? The Case for Studying Formula Composition

**DOI:** 10.1097/CCE.0000000000001465

**Published:** 2026-08-25

**Authors:** Michael J. Morowitz, Dennis M. Simon, David T. Huang, Enid E. Martinez, Nilesh M. Mehta

**Affiliations:** 1 Department of Surgery, University of Pittsburgh School of Medicine, Pittsburgh, PA.; 2 Department of Critical Care Medicine, University of Pittsburgh School of Medicine, Pittsburgh, PA.; 3 Department of Anesthesiology, Critical Care and Pain Medicine, Boston Children’s Hospital, Boston, MA.; 4 Harvard Medical School, Boston, MA.

**Keywords:** critical illness, dietary fiber, dysbiosis, enteral nutrition, formula composition, short-chain fatty acids

## Abstract

Large randomized controlled trials testing the quantity and timing of enteral nutrition (EN) in critically ill patients have consistently failed to demonstrate benefit. We propose that these negative results may partly reflect an unexamined variable: the composition of EN formulas themselves. Most widely used formulas rely on corn-derived simple carbohydrates, isolated functional fibers, dairy-based proteins, and omega-6-rich vegetable oils, ingredients selected largely for manufacturing efficiency and shelf stability rather than clinical evidence. These components bear little resemblance to whole foods, and their distinct effects on metabolism, inflammation, and gut function have received little study in critical illness. Commercially available whole-food and plant-based EN formulas now offer compositionally distinct alternatives, yet no rigorous trials have compared them to conventional formulas in critically ill patients. We argue for a paradigm shift from volume-based to composition-focused nutrition research, pairing patient-centered clinical outcomes with mechanistic endpoints, including effects on the gut microbiome.

**KEY POINTS**

- Large randomized trials testing the quantity and timing of enteral nutrition in critically ill patients have consistently failed to demonstrate benefit, and some have signaled harm.
- These trials have left one variable essentially unexamined: the composition of the formulas themselves, which are ultraprocessed and built largely on corn-derived simple carbohydrates, isolated functional fibers, dairy proteins, and omega-6-rich oils selected for manufacturing efficiency and shelf stability rather than clinical evidence.
- Compositionally distinct whole-food and plant-based enteral formulas are commercially available, yet none has been rigorously compared with conventional formulas in critically ill patients of any age.
- Critical care nutrition research should expand beyond quantity and timing to test formula composition directly, pairing patient-centered clinical outcomes with mechanistic endpoints including microbiome structure, short-chain fatty acid production, and gut barrier function.

Pick up any standard enteral nutrition (EN) formula used in ICUs and read the ingredient label. The top ingredients after water in several widely used formulas are corn syrup solids and corn maltodextrin, a highly processed glucose polymer with a glycemic index comparable to pure glucose. In one formula advertised as sole source nutrition for tube-fed children, corn maltodextrin accounts for most of the 33 grams of carbohydrate per serving while dietary fiber contributes less than 1 gram. Across some of the most used EN formulas for critically ill patients (**Table 1**), ultraprocessed, rapidly absorbed simple carbohydrates are the caloric foundation. We use the term “ultraprocessed” as defined by the NOVA classification: industrial formulations built largely from substances extracted or derived from foods (such as hydrolyzed proteins, modified starches, and corn syrup solids) together with additives, containing little or no intact whole food (1). The source of macronutrients gets little attention; for decades, the critical care community has focused instead on the amount and timing of delivery. This oversight may help explain why large randomized controlled trials (RCTs) testing different quantities or timing of nutrition, including NUTRIREA-2, TARGET, EDEN, and PermiT, have consistently failed to demonstrate benefit, and in some cases have demonstrated harm (2–5).

**TABLE 1.** Composition of Commonly Used Enteral Nutrition Formulas

Product	Primary Carbohydrate	Fiber Content/Source	Primary Protein	Primary Fat
Ensure Original	Corn maltodextrin, sugar	1 g (cellulose)	Milk protein concentrate, soy protein isolate	Canola oil, corn oil
PediaSure Enteral 1.0	Corn maltodextrin, sugar	< 1 g	Milk protein concentrate, soy protein isolate	High oleic safflower oil, soy oil, MCT
Jevity 1.5 Cal	Corn maltodextrin, corn syrup solids	FOS, oat fiber, soy fiber	Sodium caseinate, soy protein isolate	Canola oil, corn oil, MCT
Osmolite 1.5 Cal	Corn maltodextrin	None	Sodium caseinate, soy protein isolate	High oleic safflower oil, canola oil, MCT
Peptamen	Maltodextrin	None	100% hydrolyzed whey	MCT (70%), soybean oil
Isosource 1.5 Cal	Corn syrup	Pea fiber, FOS, inulin	Sodium caseinate, soy protein isolate	Canola oil, MCT
Compleat Original	Brown rice syrup	Pea fiber, FOS, inulin	Milk protein concentrate, chicken, pea protein	Canola oil
Compleat Organic Blends	Sweet potato puree, pear puree, brown rice flour	Whole-food fiber from fruits and vegetables	Hydrolyzed pea protein, rice protein	Olive oil, canola oil
Kate Farms Standard 1.0	Organic brown rice syrup solids	Organic agave inulin	Organic pea protein	Organic high oleic sunflower oil
Liquid Hope	Organic garbanzo beans, green peas, brown rice, quinoa	9 g whole-food fiber (legumes, vegetables)	Organic pea protein, almond butter	Organic flax oil

FOS = fructooligosaccharides, MCT = medium-chain triglycerides.

Ingredients listed in order of prominence on product labels. Information obtained from product labels and manufacturer websites accessed February 2026.

A recent editorial in *Critical Care Medicine* called for a more coordinated research agenda in pediatric critical care nutrition, including RCTs comparing EN formulations and integration of gut microbiome research into nutrition studies (6). We agree, and we argue that it should extend to patients of all ages. This commentary draws on both adult and pediatric critical care; although formulas and microbiome considerations differ by age, the compositional principles apply broadly. Specifically, we propose that the persistent failure of ICU nutrition trials may partly reflect an unexamined variable: the composition of EN formulas and their interaction with the gut microbiome.

Prior to the 1970s, EN was typically provided by blenderized whole foods. The shift to chemically defined formulas in the 1970s was driven by legitimate priorities (sterility, safety, nutrient standardization, controlled osmolarity, manufacturing feasibility, and cost) alongside an evolving understanding of metabolic risk. These were reasonable goals; nonetheless, we now have the tools and evidence to ask whether composition itself deserves reconsideration. Carbohydrates in most commercial EN formulas are predominantly corn-derived and simple (corn maltodextrin, corn syrup, or corn syrup solids), rapidly absorbed glucose polymers that bear little resemblance to the complex carbohydrates in whole foods. Rapid absorption is not inherently undesirable and may suit some situations, but continuous tube feeding differs from the bolus oral loads in which glycemic index is measured, and the optimal carbohydrate source remains largely unstudied. Awareness of composition is itself uneven: while many dietitians and nutrition specialists on ICU teams are well-informed, nutrition education for physicians and nurses in critical care is variable and often limited, reflecting a systemic gap in medical training rather than any individual failing (7).

Fiber’s physical characteristics (solubility, viscosity, and fermentability) determine whether it reaches the colon intact to serve as substrate for commensal bacteria, which ferment it into short-chain fatty acids (SCFAs) that support the gut barrier and modulate immunity. These properties matter in critical illness, when the gut barrier and microbiome are under stress, yet they are at least partially degraded by the heat, high pressure, and hydrolysis used to manufacture isolated functional fibers (8). Compared with whole foods, commercial EN formulas are extremely low in dietary fiber, typically less than 5 grams per 1000 kcal vs. the 14 grams per 1000 kcal recommended for healthy adults. Some formulas contain no added fiber at all, and the fiber content of the remainder varies widely (Table 1). When fiber is added, it is typically derived from isolated functional fibers such as fructooligosaccharides, oat fiber, soy fiber, pea fiber, or inulin rather than the intact fiber found within whole foods, and these isolated fibers may not fully reproduce the biological complexity or effects of intact whole-food fiber matrices. They are not inert. A recent scoping review found that prebiotic fibers such as inulin can alter the gut microbiota and increase bifidobacteria and SCFA production, but the effects are inconsistent, fiber-specific, and dose- and duration-dependent, and rarely translate into measurable clinical outcomes in critically ill patients (9). The 2016 American Society for Parenteral and Enteral Nutrition guidelines advised against the routine use of fiber-containing formulas during the acute phase while endorsing fermentable soluble fiber, such as fructooligosaccharides. This was a tepid recommendation that likely reflected fiber’s role in mitigating diarrhea—a symptom-management concern distinct from a nutritional outcome (10). The optimal fiber content and type for these patients remain unknown.

A similar blind spot exists for protein. Standard EN recommendations focus almost exclusively on protein quantity, typically targeting 1.2–2.0 grams per kilogram per day, while paying little attention to the fact that different protein sources vary markedly in their metabolic effects. Widely used EN formulas rely variously on milk protein concentrate, soy protein isolate, sodium and calcium caseinates, or hydrolyzed whey (Table 1), yet few studies have examined the type and source of protein or its impact on critical illness outcomes, and to our knowledge none has directly compared milk- and nonmilk-derived sources in ICU patients (11, 12), precisely the gap motivating this call for research. Dairy-derived proteins offer real advantages: high leucine content supporting muscle protein synthesis, complete amino acid profiles, improved digestibility of hydrolyzed forms, low cost, and standardization. These are legitimate strengths that nonetheless should not preclude investigation of alternatives. Plant-based pea and soy proteins have shown anti-inflammatory potential (reduced C-reactive protein and interleukin-6), although only in chronic disease, not in the ICU (13), and concerns about pro-inflammatory A1 beta-casein in dairy formulas add further rationale for studying nondairy alternatives (14). Whether such differences yield benefits in acute critical illness is an untested hypothesis warranting study.

Fat composition represents a fourth area of neglected variability. Standard EN formulas use varying combinations of canola oil, corn oil, high oleic safflower oil, soybean oil, and medium-chain triglycerides (Table 1). These blends are rich in omega-6 fatty acids, particularly linoleic acid, the dietary precursor of arachidonic acid and its pro-inflammatory eicosanoid derivatives, raising concern about delivering high omega-6 loads to patients already experiencing systemic inflammation. The omega-3 literature shows how inattention to fat composition can confound trials: early positive studies used omega-6-rich comparators, potentially rendering controls pro-inflammatory, while the OMEGA trial with a neutral control showed no benefit (15), even as a later meta-analysis supported reductions in ICU length of stay and ventilation with omega-3 supplementation (16). As with carbohydrate and protein, the fat profiles of current formulas reflect manufacturing decisions based on scant clinical evidence.

The composition of EN macronutrients is not merely a theoretical concern; emerging evidence suggests it may have measurable consequences for the gut microbiome, increasingly recognized as central to critical illness pathophysiology. Critical illness induces profound dysbiosis characterized by loss of microbial diversity and overgrowth of potentially pathogenic taxa (17, 18). Less often considered is the possibility that our nutritional interventions are themselves contributing to this problem. The impact of macronutrient source on the microbiome may be a missing link, a hypothesis that motivates the mechanistic agenda below.

Complex carbohydrates and dietary fiber are fermented by commensal bacteria into SCFAs such as butyrate, which maintain intestinal epithelial integrity, promote regulatory T-cell differentiation, and suppress systemic inflammation (19). When these substrates are replaced with corn maltodextrin and sugar, which are absorbed in the proximal small intestine and never reach the colonic microbiota, beneficial bacteria are deprived of their primary fuel source. In this light, the dysbiosis observed during critical illness may be partly iatrogenic, driven not only by antibiotics and illness severity but also by the composition of the formulas we deliver. Preliminary data from our group and others suggest associations between enteral formula composition and microbial community structure in critically ill patients (17, 18). These findings are especially hard to interpret in children, whose developing microbiome shifts rapidly with age, compounding the challenge of isolating formula effects in critically ill pediatric patients. Trial designs must account for this complexity.

Efforts to manipulate the microbiome in critical illness are not new: probiotics, synbiotics, and prebiotic fibers have been tested in the ICU, with some trials suggesting reductions in ventilator-associated pneumonia, diarrhea, and length of stay. Yet the evidence is heterogeneous in strain, dose, and duration, and recent guidelines offer only weak recommendations based on low-certainty evidence (20). These mixed results are part of our rationale: rather than adding a single microbial agent or fiber atop an ultraprocessed base formula, modifying the nutritional substrate itself is a complementary, largely unexplored strategy worth pursuing alongside direct microbial manipulation.

To be clear, conventional EN formulas were a major advance in supportive care and have sustained millions of critically ill patients who could not eat by mouth. Their design also reflects genuine patient-safety imperatives (minimizing contamination, maintaining shelf stability and nutrient consistency after opening, avoiding tube occlusion, and managing allergens) that created real tradeoffs. As we reconsider composition, the question is not whether safety mattered but how more whole-food-based formulations can manage these constraints while delivering compositional diversity. Several products listed in Table 1 demonstrate that whole-food EN formulations are not hypothetical; they are commercially available and in use, delivering phytochemicals, polyphenols, and micronutrients in their native food matrix. These differences warrant investigation but are beyond the scope of this commentary. Their advantages should not be overstated, however: important questions remain about tolerability, protein quality and completeness, viscosity and tube compatibility, cost and batch-to-batch variability, and, most fundamentally, whether their compositional differences yield meaningful microbiome or clinical benefits in acute critical illness. That conventional formulas have saved lives does not mean they cannot be improved; the case for alternatives must be settled by rigorous trials, not assumed. The purpose of this commentary is to highlight this important and missing line of investigation in nutrition during critical illness.

Future trials should compare different EN compositions head-to-head while incorporating mechanistic endpoints: microbiome diversity through longitudinal stool sampling, gut barrier function via biomarkers such as zonulin and fecal calprotectin (21), SCFA profiles as a functional readout of microbial metabolism, and patient-centered clinical outcomes. The precedent exists outside critical care, where a microbiota-directed complementary food-2 reshaped the gut microbiome and improved growth in undernourished Bangladeshi children (22).

In conclusion, we propose a fundamental shift in critical care nutrition research toward the source of macronutrients. The ingredients in ICU nutrition formulas are not pharmacologically inert; they are biologically active compounds that shape the gut microbial ecosystem, influence intestinal barrier function, and modulate systemic inflammation. The time has come to study them with the same rigor we apply to medications, ventilator settings, and fluid management, testing composition alongside the quantity and timing of nutrient delivery, with collaboration among critical care, nutrition science, and microbiology. The key to unlocking the elusive nutrition-outcome relationship may be hiding in plain sight, on the ingredient label.
